# Verification of *Meso*-Zeaxanthin in Fish

**DOI:** 10.4172/2157-7110.1000335

**Published:** 2014-06-01

**Authors:** John M Nolan, Stephen Beatty, Katie A Meagher, Alan N Howard, David Kelly, David I Thurnham

**Affiliations:** 1 Macular Pigment Research Group, Department of Chemical and Life Sciences, Waterford Institute of Technology, Waterford, Ireland; 2 Howard Foundation, Cambridge, UK; 3 Northern Ireland Centre for Food and Health (NICHE), University of Ulster, Coleraine, UK

**Keywords:** Meso-zeaxanthin, Lutein, Zeaxanthin, HPLC, Carotenoids, Fish, Lutein, Zeaxanthin, *Meso*-zeaxanthin, Macular pigment, Fish, Seafood, Food analysis, Food composition

## Abstract

**Background/Objectives:**

The carotenoids lutein (L), zeaxanthin (Z), and *meso*-zeaxanthin (MZ) accumulate in the central retina (the macula), where they are collectively known as macular pigment (MP). MP has been shown to enhance visual function in both diseased and non-diseased retinae, and therefore an understanding and confirmation of, the origins of these carotenoids is needed. Studies have shown that L and Z are present in many foodstuffs found in a typical Western diet (e.g. spinach, kale, peppers, yellow corn and eggs). It has been shown that MZ is generated from L in the primate retina and earlier reports suggested that MZ was present in some fish species. Recently, however, one research group reported that MZ is not present in fish and suggested that the earlier reports showing MZ in these marine species were a methodological artefact. The current study was designed to investigate the reason for the contradiction, and test for the presence of MZ in fish and some other foods.

**Methods:**

Raw fruits, vegetables and fish were extracted for carotenoid analysis by high performance liquid chromatography.

**Results:**

MZ was not detected in any of the fruits or vegetables tested in our study. However, using retention time matching, absorption spectrum comparison, and sample spiking, we verified the presence of MZ in salmon skin, sardine skin, trout skin and trout flesh.

**Conclusion:**

This study confirmed the presence MZ in nature, and in the human food chain.

## Introduction

The carotenoids are a family of tetraterpenoid compounds responsible for the vivid colours of many fruits, vegetables, and flowers [1] Over 700 carotenoids have been identified to date, more than 40 of which are found in fruits and vegetables. Despite their abundance in nature, only 14 of these compounds can be absorbed by the human body [2]. Three hydroxycarotenoids, lutein (L), zeaxanthin (Z) and *meso*-zeaxanthin (MZ), accumulate at the macula (to the exclusion of all other dietary carotenoids), where they are collectively known as macular pigment (MP) [3-5].

The macula is a specialized part of the retina, which facilitates central and colour vision. The biological selectivity whereby L, Z and MZ accumulate at the macula is likely to be non-coincidental, and to have evolved in response to the functional needs of the central retina [6]. Indeed, MP is a short wavelength (blue) light filter [7] and a powerful antioxidant, [8,9] and it has been shown that enrichment of MP in both the non-diseased retina (i.e. in subjects free of retinal disease [e.g. drivers, sports people etc.] [10-12] and the diseased retina (i.e. in patients with age-related macular degeneration) can enhance visual function and comfort in these individuals [13,14].

L is the dominant carotenoid in the peripheral macula, Z in the mid peripheral macula, and MZ at the epicentre of the macula. L and Z are entirely of dietary origin [15-17] while MZ is thought to be derived (at least in part) from retinal L [18,19], through a poorly-understood process of bioconversion. Indeed, the hypothesis that retinal MZ is derived from retinal L is supported by only two studies, one in Rhesus monkeys [18] and one in quail [19]. MP scientists routinely cite MZ as “non-dietary”, even though its presence in 20 fish species has been previously reported by Maoka et al. in 1986, [20] but its presence in the Western diet has not been comprehensively studied to date. This lack of data on MZ in foods is likely due to the challenges inherent in the separation and quantification of this carotenoid from foods [21].

These experiments were conducted to analyse foods, known to contain L and Z, for the possible presence of MZ. Also, we analyzed several fish species for MZ, as most previous publications have reported MZ to be present in these marine species [20-23]. It is interesting to note that these authors suggest MZ is a reductive metabolite of *meso*-astaxanthin in fish, however, a recent study by Rasmussen et al concluded that MZ was not present in any of the fish species tested in their study [24]. In addition the authors suggested that the results obtained by Maoka and others were an artefact that may have been produced by the methods used in their experiments. In summary, the experiments performed in the current report were conducted to clarify the following important research question. Is MZ present in food or is it unique to the macula? We also investigated the reason for the recent contradiction of the previous reports of MZ in fish species.

## Materials and Methods

### Standards and solvents

**Figure 1:**
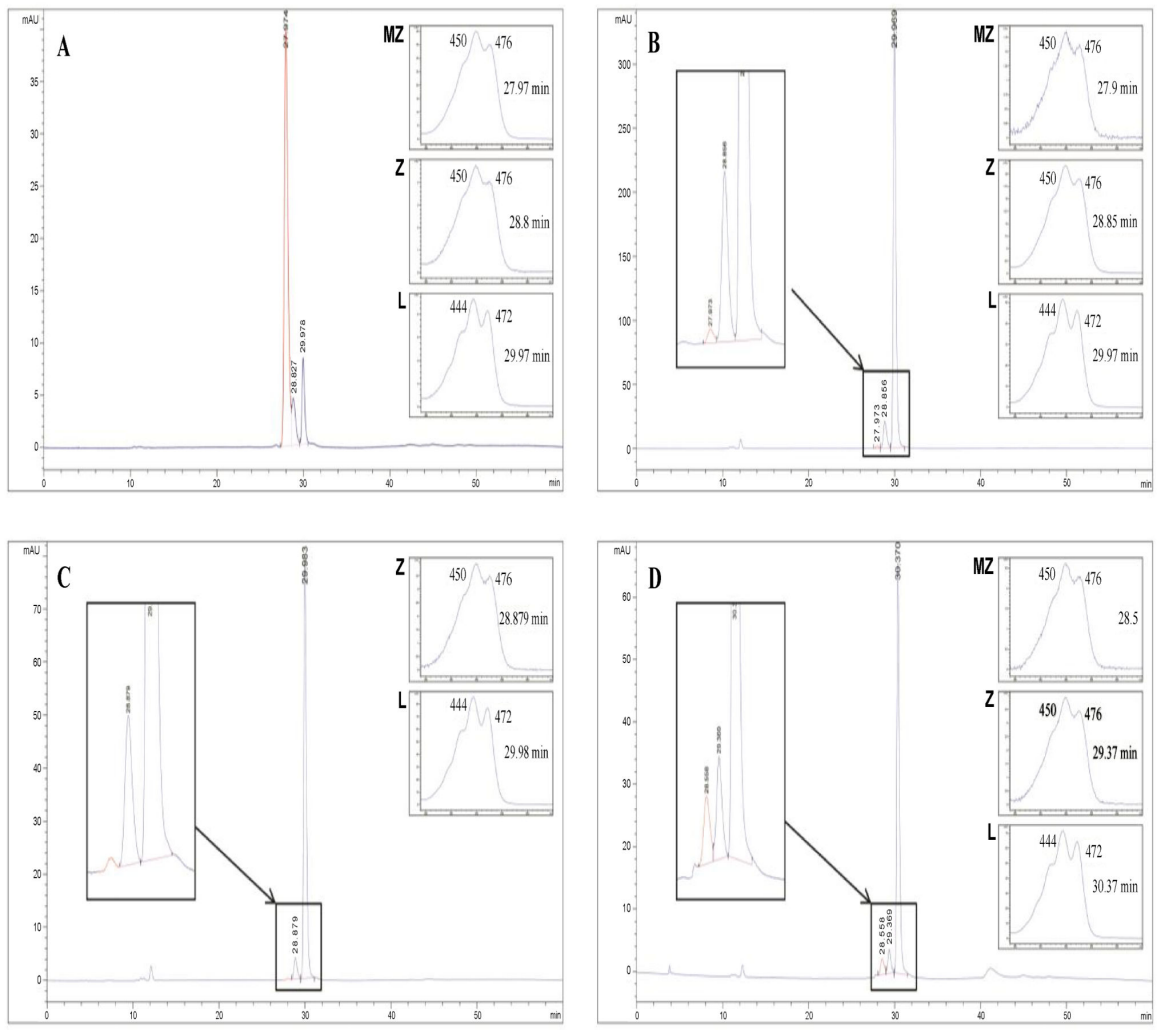
Saponification of Lutein under different saponification conditions MZ = meso-zeaxanthin (highlighted in red); Z = zeaxanthin; L = lutein; Normal phase chiral conditions were used to obtain the above chromatographs. Absorbance spectra are shown for each peak with the exception of MZ in chromatograph C (no absorption spectrum was obtained for this small MZ peak). MZ peak highlighted in red. A. illustrates a standard mixture of MZ, Z and L which was used for retention time matching as part of MZ identification (see retention times and absorption spectra); B. illustrates a non-saponified L standard; C. illustrates the L standard after mild saponification (5% KOH at 45°C overnight); D. illustrates the L standard after intense saponification (10% KOH at 120°C overnight); Note: only following intense saponification of the standard was L converted to MZ, reflected in an altered MZ:L %ratio

DSM Nutritional Products supplied the L and Z reference standards. The MZ standard was supplied by Industrial Organica as a soya bean oil oleoresin (Figure 1A). All solvents (high performance liquid chromatography [HPLC] grade) used for extraction and HPLC analysis were supplied by Sigma-Aldrich.

### Foods analyzed

The following foods were analysed as part of these experiments:

#### Vegetables

*Capsicum annuum* (yellow and orange peppers), *Spinacia oleracea* (spinach), *Cucurbita moschata* (squash), *Phaseolus vulgaris* (green beans), *Brassica oleracea* (broccoli), *Zea mays* subsp. *Mays* (maize or corn);

#### Fruits

*Citrus × sinensis* (orange), *Carica papaya* (papaya), *Cucumis melo var. cantalupensis* (cantaloupe), *Mangifera indica* (mango), *Malus domestica* (apples), *Actinidia deliciosa* (kiwifruit), *Musa acuminata* (bananas), *Fragaria × ananassa* (strawberries);

#### Fish

(flesh and skin): *Salmo salar* (salmon), *Oncorhynchus mykiss* (trout), *Sardina pilchardus* (sardine), *Scomber scombrus* (mackerel), *Pleuronectes platessa* (plaice).

Fruits and vegetables were sourced from local supermarkets in the Waterford region (South East of Ireland). All fruits and vegetables were analyzed within their declared expiry dates. Fresh fish were sourced from a local fish monger (Billy Burke’s Fish Shop BallyBricken, Waterford Ireland), and were traced back to their origin (i.e. wild-caught or farmed). The origin of fish analyzed in this study can be summarized as follows: Farmed salmon was sourced from Wester Ross, Scotland, United Kingdom and the farmed trout was sourced from Goatsbridge, Co. Kilkenny, Ireland; sardine, mackerel and plaice were wild-caught fish.

#### Sample preparation

Raw fruits, vegetables and fish were analyzed for xanthophyll carotenoids according to the following methods. The raw foodstuff was segmented, and the inedible portions removed. For fish samples, the skin was removed from the flesh and tested separately. The remaining edible portions (circa 5 to 20 g depending on food type) were homogenised in a blender (Waring Blender, 1L, Waring, The Conair Group Ltd, Fleet, UK) with a small quantity of water (20-40 mL, depending on food type) for vegetables and fruits (and in the case of fish samples with an additional equivalent volume of acetone) for no more than 5 minutes to produce a homogenous slurry.

A sample of 5 to 10 g of this slurry was removed to a Duran bottle and mixed with 20 mL of the extraction solvent, consisting of a 1:1 mixture of di ethyl ether/petroleum spirit (40 to 60°C boiling point). The mixture was agitated using magnetic stirring for 30 minutes to an hour to adequately extract the carotenoids and break the emulsion that formed on addition of the extraction solvent mixture. The resulting organic layer (circa 15 mL) was removed and equally divided between 2-3 sample vials and dried under nitrogen. This allowed 2-3 samples for the saponification experiment i.e. one non-saponified sample and at least one saponified sample from the same extract. For fish samples, a third sample aliquot was retained for strong saponification, as described below.

#### Saponification of lutein standard

To investigate the impact of saponification on L, and in order to address the possibility that MZ may be artefactually generated (from L) during the saponification process, we subjected a L standard to mild, strong and intense saponification processes, as follows: mild saponification (5% aqueous [potassium hydroxide [KOH] at room temperature overnight); strong saponification (10% aq. KOH at 45°C overnight); intense saponification (10% aq. KOH at 120°C [in an oven] overnight).Of note, the L standard (provided by DSM) contained MZ:Z:L in a ratio of 1:13:154.

#### Food sample saponification

Vegetables, fruits and fish samples were saponified using differing saponification conditions, because of dissimilarities in sample matrix between food types. For all foods tested, the first sample aliquot was analysed without saponification (for the purposes of comparison). Vegetables and fruits were saponified with a 4% KOH concentration only, while fish samples were also subjected to mild (5% KOH) and strong (10% KOH) saponification conditions. The samples intended for saponification were each dissolved in 10 mL of ethanol, and an appropriate volume of 25% aqueous KOH was added to obtain the final required KOH concentrations, as above. These samples were housed in a shaking incubator (Stuart Incubator SI500, Dublin, Ireland) at 45°C overnight. The samples were then removed and allowed to cool. An equivalent aliquot of the extraction solvent mixture (a 1:1 mixture of di ethyl ether/petroleum ether) was added to the samples and the resulting mixture was agitated using magnetic stirring. The organic upper layer was then removed to a separate Duran bottle and washed with water (circa 20 mL). This washing step was repeated twice. In the case of fish samples, this washing step was combined with a salting out step using approximately 5 g of anhydrous sodium sulphate to remove water. The samples were dried under nitrogen and prepared for HPLC analysis immediately after extraction.

#### HPLC analysis

Vegetable and fruit extracts (non saponified and saponified) were reconstituted in the appropriate mobile phase in accordance with the HPLC separations: 1 mL of acetonitrile/methanol/triethylamine, 85/15/0.1% for reverse phase separation (Assay 1) and 1 mL of hexane/isopropanol, 90/10, v/v for normal phase separation (Assay 2). Fish flesh and fish skin extracts were not soluble in the mobile phase of Assay 1 and therefore these extracts were analysed directly using Assay 2.

For Assay 1, the reverse phase separation was carried out using a Phenomenex Ultracarb ODS (20) 3 μm C18 column, 250×4.6 mm with a guard column (Phenomenex, Cheshire, UK) and a 0.5 μm inline filter (Upchurch; Sigma-Aldrich). The method used a premixed isocratic mobile phase consisting of 85% acetonitrile, 15% methanol and 0.1% triethylamine. The flow rate was maintained at 1 mL/min for the duration of the 40 minute run.

For Assay 2, the normal phase separation was carried out using a 3μm chiral column (Chiralpak IA-3 column [250 × 4.6 mm]), a guard column and 2 μm filter (both sourced from Apex Scientific Limited, Kildare, Ireland). The gradient method consisted of an initial 90:10 n-hexane:isopropanol mixture, which increased to 85:15 over 20 minutes and plateaued at this ratio for 5 minutes. The system was allowed to return to initial settings over a 5 minute time period, completing the 30 minute run time. The presence of MZ was verified by standard-matched retention time (Figure 1A), absorbance spectra, and by co-elution (spiking) with an MZ standard.

Ester peaks (seen in unsaponified samples) were confirmed using Ultraviolet-visible (UV/VIS) spectroscopy, as indicated by the presence of a large peak at 292 nm in conjunction with the normal carotenoid absorbance spectra.

## Results

### Saponification experiment

Figure 1A illustrates a standard mixture of L, Z and MZ. Figure 1B illustrates a non-saponified L standard. Saponification of the L standard using mild (5% KOH at room temperature overnight, see Figure 1C) and strong (10% KOH at 45°C overnight) processes did not generate MZ from L, whereas using intense saponification conditions (10% KOH at 120°C overnight) does generate MZ from L (Figure 1D), reflected in a 10-fold increase in the amount of MZ (Table 1).

### Vegetables and fruits

MZ was not detected in any of the fruits or vegetables analysed in this experiment (for either saponified or non-saponified samples).

### Fish

**Figure 2:**
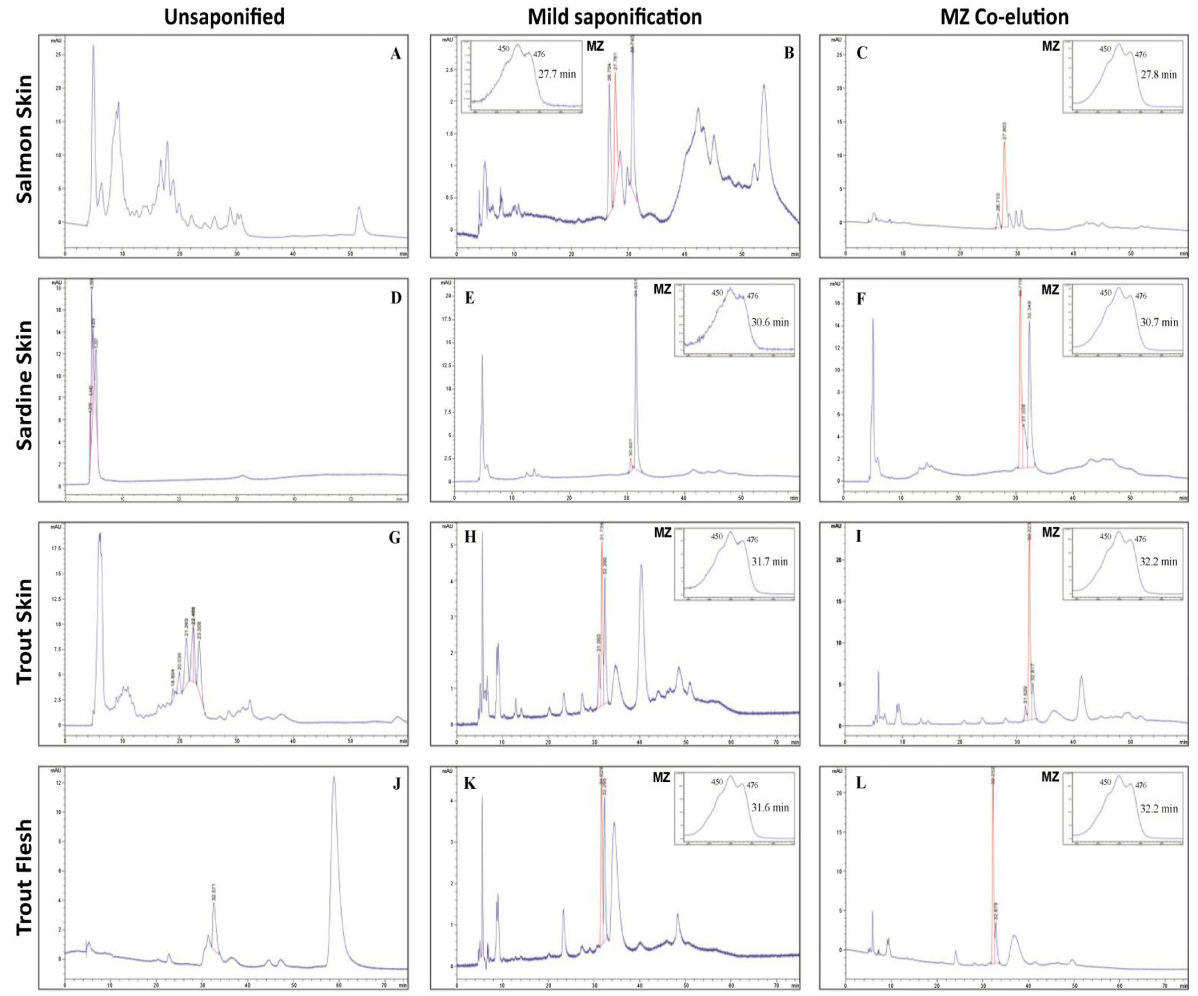
Chromatography illustrating the presence of MZ in several fish species tested MZ = meso-zeaxanthin (highlighted in red); Z = zeaxanthin; L = lutein; Normal phase chiral conditions were used to obtain the above chromatographs. Absorbance spectra are shown for the MZ peak. A, D, G and J = chromatography for non-saponified samples showing ester peaks obtained from salmon skin, sardine skin, trout skin, and trout flesh, respectively; B, E, H and K = chromatography for saponified samples (5% KOH at room temperature overnight) showing MZ peaks obtained from salmon skin, sardine skin, trout skin, and trout flesh, respectively; C, F, I and L = chromatography for saponified samples (5% KOH at room temperature overnight) co-eluted (spiked) with MZ standard showing increased MZ peaks obtained from salmon skin, sardine skin, trout skin, and trout flesh, respectively

A peak with matched retention time to an MZ standard (circa 30 mins) and exhibiting MZ spectrophotometric characteristics was identified in the following foods: salmon skin (Figure 2B, 27.7 mins), sardine skin (Figure 2E, 30.6 mins), trout skin (Figure 2H, 31.7 mins) and trout flesh (Figure 2K, 31.6 mins). Of note, using Assay 2 the retention time shifts slightly from run to run and therefore a standard was run with each sample to ensure the accuracy of retention time matching. These findings were corroborated by co-elution with a MZ standard (Figure 2C, F, I and L). Of note, the MZ peak was only present when the fish samples were saponified using 10% KOH at 40°C overnight. In the non-saponified samples (Figure 2A, D, G and J), esters were present, but as the peaks were not resolved, these esters could not be identified.

## Discussion

These experiments confirmed the presence of MZ in the skin and flesh of certain fish species, whereas MZ was not detected in the fruits or vegetables analysed. Of note, the paucity of data available on foods containing MZ is probably because: 1. the importance of MZ has only recently been identified, and MZ is therefore a relatively new compound of interest to researchers and 2 there are challenges inherent in the separation and quantification of MZ [9,10,25,26]. The majority of analytical HPLC methods used to analyse carotenoids are not designed to separate MZ from Z, and, therefore, have reported total Z [MZ and Z] concentration, as chiral chromatography is required to separate these isomers). However, improvements in analytical instruments (e.g. nuclear magnetic resonance, mass spectroscopy, HPLC, etc.) have made it possible to isolate and identify new carotenoids in nature, including MZ [21,27-29].

Furthermore, given that carotenoid analysis from foods is problematic and time-consuming, [30] it is important to use appropriate extraction methods to ensure valid analysis. For example, it is important to saponify foods in a way that allows the carotenoids to be released from the food matrix, [31] yet does not destroy the carotenoids. Our experiments showed that saponification is, indeed, required to separate MZ from fatty (esterified) samples, as the non-saponified fish samples exhibited carotenoid ester peaks in the chromatographs (confirmed by UV/VIS spectroscopy), but these eluted as clustered peaks, which are not suitable for carotenoid identification [32]. In other words, in order to accurately identify MZ, it is necessary to hydrolyse carotenoid esters using saponification when analysing fish samples, since xanthophylls are typically esterified in fish [22,23].

Our observation that MZ is present in fish is consistent with previous reports of several authors [20,22,23]. These authors assumed that MZ was a reductive metabolite of meso-astaxanthin in fish since they detected MZ in many marine species (e.g. Maoka et al: salmon, rainbow trout, black bass, red halibut; Schiedt et al: rainbow trout; Katsuyama et al: rainbow trout and tilapia). In contrast, a very recent study [24] concluded that L, Z and MZ were not present in any of the fish or seafoods tested in their study (e.g. salmon, sea bass, trout, bluefish, shrimp). However, it is important to point out that the investigators did not saponify any of the samples tested, and therefore would not have been able to detect free form L, Z or MZ using their method (as indicated above).

Dietary carotenoids are known to play an important role in colour regulation of fish skin and muscle. These carotenoids and their resulting colours also play a role in mating and spawning of fish, and are also believed to be important for camouflage. Fish are less visible in deep water which is impenetrable to long [red] wavelengths of the visible spectrum [33,34]. Fish do not possess the ability to biosynthesize carotenoids *de novo*, but they can modify dietary carotenoids stored in the integument and other tissues. For example, it has been shown that astaxanthin, which is commonly used as a feed supplement in fish farming, is metabolised to MZ in rainbow trout, salmon and tilapia fish [22,23]. Of note, farmed fish have no access to naturally occurring carotenoid-rich feed, and hence the need for supplementation with these compounds. From a consumer’s perspective, pigmentation is an important attribute of edible fish, and is therefore important for its market value. Beyond conferring a desirable colouration, evidence for beneficial biological actions of carotenoids in fish is emerging. For example, fish supplemented with carotenoids (e.g. astaxanthin) have been shown to exhibit lower concentrations of serum lipid peroxides, reduced susceptibility of liver to lipid oxidation, and protection against environmental hazards (e.g. light, temperature, low oxygen tension ammonia). Moreover, carotenoid supplementation has been shown to enhance fish growth and protect against disease [35-37]. Similarly, in humans, there is emerging evidence that carotenoids may be important for optimal health. They are believed to protect against certain cancers e.g. lycopene may protect against prostate cancer, and the antioxidant and light-filtering properties of the xanthophylls (L, Z, and MZ) are known to protect against progression of AMD [13,38-42].

Of note, in the current study, MZ was found in the skin (only) of 3 of the 4 species of fish that we examined, whereas in trout this carotenoid was also identified in the flesh. Although the skin is often removed from freshly cooked fish, [43] fish skin is typically retained for consumption in canned fish (e.g. sardines, salmon and mackerel) and pickled fish (e.g. herring, trout, salmon, mackerel). In addition, many restaurants choose to serve fish with the skin, as it is believed to lock in the flavours. The MZ identified in trout flesh in our experiment may be due to the farmed nature of the trout tested but it is nonetheless important to note that MZ is in fact found in the human food chain. Supplementing farmed fish with astaxanthin is a practice common to many fish farms in order to ensure attractive colouration of the product, and MZ is a known metabolite of astaxanthin in such fish. Thus, these experiments confirm the presence of MZ in the human food chain [22,35,37,44].

Of note, in our study, we did not detect MZ in any of the fruits or vegetables tested. As mentioned above, it is possible that the source of MZ in the salmon, trout, and sardines may well have been astaxanthin. In this study, both salmon and trout analyzed were produced in fish farms and it is known that astaxanthin or canthaxanthin will have been provided in the diets for these fish. Also, trout and salmon are closely related and in the same genera (Onchorhynchus) and may metabolise carotenoids in a similar fashion to each other. In contrast, we found no MZ in the two wild-caught samples of mackerel and plaice and it is therefore tempting to speculate that this was because they did not receive supplemental astaxanthin as a predominant carotenoid, but instead obtained a mixture of carotenoids from natural food sources to provide their skin pigmentation. Also, sardines are in a different family from all the other fish tested. Interestingly, they were not farmed and therefore not supplemented with astaxanthin, but their natural diet may have contained the necessary carotenoids to provide MZ in these fish species. One of the important findings emerging from this study is that the presence of MZ in sardines confirms the presence of MZ in the human food chain, even in the absence of supplementation with astaxanthin, since sardines are not farmed like trout or salmon.

Having identified MZ in fish in this qualitative study, it is now incumbent upon the scientific community to investigate the concentrations of this carotenoid in those and other species. Such a study will not be without challenges beyond those required for a qualitative study, and these include: identification and validation of an internal standard suitable for different fish species when analysing MZ; the high lipid content of the sample (fish), which renders extraction problematic; the esterified nature of the carotenoid will necessitate a saponification process, which will inevitably result in loss of some of the carotenoid being quantified; difficulties presented by the nature of the fish matrix for which MZ content is being investigated, because such a matrix does not lend itself to analysis under usual HPLC conditions used to quantify macular carotenoids (including MZ).

In conclusion, this was a preliminary study to investigate why workers in a recent report [24] had not detected MZ in fish species, even though several previous reports [20,22,23] had shown it to be present. We showed that mild and strong saponification did not artefactually produce MZ from L and that strong saponification was necessary to hydrolyse xanthophyll esters in fish products. As expected, we did not detect MZ in any of the fruits or vegetables tested, but we detected MZ in salmon skin, sardine skin, trout skin and trout flesh, and therefore confirmed its presence in these foods. These findings support the hypothesis that retinal MZ may not be derived wholly and solely from retinal L, as MZ may be consumed (albeit in small quantities) as part of a human diet. Having detected the presence of MZ, we now need to quantify the amount of MZ present in fish products and the human diet.

## Figures and Tables

**Table 1:** Percentage ratios of MZ, Z and L under different saponification conditions

Carotenoid ratio %	Standard (unsaponified)	Mild saponification	Strong saponification	Intense saponification
MZ%	0.6	0.5	0.5	5.5
Z%	7.6	6.2	6.9	7.7
L%	91.8	93.3	92.6	86.8
L:MZ	153	186.6	185.2	15.8

MZ = meso-zeaxanthin; Z = zeaxanthin; L = lutein; Carotenoid ratio % refers to the percentage of each carotenoid in the standard. Standard (unsaponified) = unsaponified lutein standard; Mild saponification = 5% KOH at room temperature overnight; Strong saponification = 10% KOH at 45°C overnight; Intense saponification = 10% KOH at 120°C overnight. Note: only following intense saponification of the standard was L converted to MZ, reflected in an altered L: MZ %ratio
